# Unbundling Strategic Change in Family Firms: the Influence of Familiness on the Strategic Change Process

**DOI:** 10.1007/s41471-021-00117-5

**Published:** 2021-10-15

**Authors:** Moritz Belling, Ulrich Pidun, Dodo zu Knyphausen-Aufseß

**Affiliations:** 1 Technische Universität Berlin, Straße des 17. Juni 135, 10623 Berlin, Germany; 2 Fachgebiet Strategische Führung und Globales Management, Technische Universität Berlin, Straße des 17. Juni 135/H 92, 10623 Berlin, Germany

**Keywords:** Family businesses, Familiness, Resource-based view, Strategic change, Gradual and radical change episodes

## Abstract

The viability and adaptation of family firms is a key research area owing to the longevity and transgenerational vision of the family. Throughout their development, firms transition through strategic change episodes with a potentially significant impact on their performance and survival. In this article, we combine family firm with strategic change research to propose how familiness supports or limits strategic change. We put forward three tendencies of family firms in their ability to deal with strategic change. First, familiness creates an overemphasis on the cognition of gradual change triggers but limits the cognition of radical change triggers. Second, familiness creates a tendency to inappropriately scope and dimension strategic change in radical change episodes to protect the value of legacy resources. Third, familiness supports endurance during strategic change implementation while also creating a tendency to be too slow or stubborn when implementing an insufficient change decision.

## Introduction

Family firms are the most common type of business firm worldwide (e.g., Faccio and Lang 2002; La Porta et al. 1999), often outperforming their peers (e.g., Anderson and Reeb 2003a; Lee 2006; Maury 2006; Martínez et al. 2007) and reaching remarkable ages. In Germany, for example, the 35 oldest family firms are all older than 250 years.0F0F^1^ This implies that family firms are a particularly viable and adaptable type of organization. Moreover, longevity is considered a key objective of the family in achieving its dynastic motive and transgenerational control intentions for the firm (e.g., Chrisman et al. 2012; Gómez-Mejía et al. 2007), which enables family firms to pursue unique strategies (e.g., Zellweger 2007) often centered around protecting the legacy business and family resources.

However, the established archetype of the long-term oriented family firm is under increased scrutiny by researchers and management practitioners. Family firms are often associated with high failure rates (e.g., Ward 2016; Wilson et al. 2013) and stagnation (e.g., Ward 1997) caused by family-internal changes such as generational transition (e.g., Aronoff 2004; Vallejo 2008; Ward 1997). Moreover, they are increasingly challenged by young, fast-growing technology companies which target traditional industry domains with disruptive technologies and business models. While incumbent family firms are often restricted by conservatism, risk aversion (e.g., Cassia et al. 2012), and the perpetuation of family legacy and traditions (e.g., DeTienne and Chirico 2013; Ogbonna and Harris 2001), these emerging technology companies can tap an ever large market for risk-seeking venture financing, as well as a seemingly infinite pool of young and motivated digital talent in the global technology hubs. Who would have thought that, for example, Tesla—a Silicon Valley based manufacturer of electric vehicles founded in 2003—would become one of the biggest threats to Germany’s incumbent and predominantly family-owned automotive industry in less than 20 years? That the traditional printing industry with its tremendously successful, often family-owned incumbents would start to diminish with the rise of new media and digital technologies? Despite those adversities, however, many family firms achieve longevity, causing high variance in family firms’ lifespans (Ciravegna et al. 2020). Even though many scholars devoted their attention to understanding family firm longevity (e.g., Ahmad et al. 2020; Kim and Gao 2013; Stamm and Lubinski 2011; Zellweger et al. 2012), explaining this “family firm longevity paradox” of lifespan variances (Ciravegna et al. 2020, p. 110) and its causes remains a key challenge for family firm researchers.

In strategic management research, the question of how firms change and adapt to survive is a central concern that has been addressed from a variety of perspectives (e.g., Ansoff et al. 2018; Chakravarthy 1982; Dosi and Nelson 1994; Kraatz and Zajac 2001; Mintzberg 1978; Nelson and Winter 2009; Rajagopalan and Spreitzer 1997; Sirmon and Hitt 2003; Spender 1996; Teece et al. 1997). A key manifestation of change and adaptation over a firm’s life span is strategic change, defined as changes in the scope, resource deployment, or competitive advantage of a firm to adapt to changing environmental requirements (Rajagopalan and Spreitzer 1997). In the strategic change process, firms take corrective actions through managerial discretion to correct a misfit and realign with their environment (Müller and Kunisch 2018). Inadequate strategic change can have detrimental effects on firm outcomes, including firm survival (Haveman 1992; Meyer et al. 1990; Tushman and O’Reilly III 1996) and performance (Klarner and Raisch 2013; Rajagopalan and Spreitzer 1997). Moreover, strategic change is a multi-step process (Kunisch et al. 2017), which requires the cognition of a need or opportunity for change (e.g., Helfat and Peteraf 2015; Johnson 1992; Ocasio 1997; Rajagopalan and Spreitzer 1997), an appropriate strategic change decision in terms of scope and dimensioning (e.g., Müller and Kunisch 2018; Rajagopalan and Spreitzer 1997), and successful implementation (e.g., Kunisch et al. 2017; Stouten et al. 2018).

Family firm research (e.g., Brunninge et al. 2007; Chirico and Salvato 2008; Drozdow and Carroll 1997; Ogbonna and Harris 2001; Ward 1997) and strategic change research (e.g., Bethel and Liebeskind 1993; Boeker 1989; Bohman and Lindfors 1998; Bourgeois III 1984; Goodstein and Boeker 1991) agree that family involvement in ownership and management influences strategic change. Within this article, we conceptualize the distinctiveness of family firms using the resource-based familiness concept, which suggests that family firms develop unique and valuable resources through the systemic interaction between family and firm (e.g., Habbershon and Williams 1999; Habbershon et al. 2003; Pearson et al. 2008; Sirmon and Hitt 2003). Familiness resources can further be segregated into different resource bundles, including, inter alia, managerial human capital, social capital, and patient financial capital (Sirmon and Hitt 2003). Despite the positive connotation of resources as a source of competitive advantage (Barney (1991), researchers have suggested that familiness can have both positive and negative effects for the firm (Habbershon et al. 2003; Weismeier-Sammer et al. 2013). Consequently, enhancing the understanding of the linkage between familiness and strategic change may help explain the family firm longevity paradox. It is also of high practical relevance since many family firms are increasingly forced to react to exogenous shocks to survive, such as the 2008 financial crisis or the recent COVID-19 pandemic.

Thus far, much of the existing research on family firms has focused on explaining why the firms engage in specific types of strategic change, such as internationalization (e.g., Pukall and Calabrò 2014), mergers and acquisitions (e.g., Gomez-Mejia et al. 2018), technological innovation (e.g., Kammerlander and Ganter 2015; König et al. 2013), new industry entries (e.g., Gu et al. 2019), or business exits (e.g. Salvato et al. 2010). However, existing research rarely incorporates a holistic and more complex set of mechanisms along the three outlined steps of the strategic change process that combine cognition, decision, and implementation.

To fill this conceptual gap, the research question in this article is as follows: *What are the supporting and limiting effects of familiness on the strategic change process?* By answering this question, our article contributes to family firm literature by extending efforts to better understand how family firms evolve and change. We propose three tendencies of strategic change in family firms, based on a process model that is agnostic on strategic change triggers and types; we then suggest a link between the presented propositions, environmental change episodes, and firm performance. Thus, we advance the understanding of the impact of familiness on firm performance (Chrisman et al. 2013; Rau 2014; Weismeier-Sammer et al. 2013) and propose that such impact is contingent on the environmental change episode.

Moreover, our article contributes to the strategic change literature by demonstrating how ownership characteristics can provide a potential explanation for the heterogeneity in the outcomes of strategic change (Müller and Kunisch 2018). On a more granular level, we develop propositions on the impact of resources on the cognition, decision, and implementation steps of strategic change. Connecting our propositions may enhance the understanding of the complex relationship between firm resources, environmental determinism, and firm outcomes of strategic change.

This paper proceeds as follows: In the next section, we summarize the existing literature on strategic change, familiness, and the influence of familiness on strategic change. In the third section, we develop propositions on how familiness influences the mechanisms along the strategic change process that we further differentiate by the environmental change episode. In the fourth section, we connect the propositions to derive three tendencies created by familiness in the strategic change process and discuss their link to firm performance. In the final section, limitations and areas for future research are presented.

## Theoretical Background

### Strategic Change

Strategic change—the change of a firm’s scope, resource deployment, or competitive advantage to adapt to changing environmental requirements (Rajagopalan and Spreitzer 1997)—is a central concern in management research and has gained increasing attention from the scholarly community over the last decades (Kunisch et al. 2017). Despite the growing relevance of the topic and diverging perspectives, a recent literature review by Müller and Kunisch (2018) identified three shared assumptions on strategic change, on which we elaborate in the following paragraphs.

First, strategic change is influenced by managers through their interpretation and decision-making sovereignty in the firm (e.g., Bohman and Lindfors 1998; Bourgeois III 1984). For example, the distinct managerial resources accumulated throughout their career influence strategic change, e.g., via managerial cognition (Helfat and Martin 2015; Helfat and Peteraf 2015). In addition, managerial actions influence the organizational resistance to strategic change (Rajagopalan and Spreitzer 1997), an essential impediment to strategic change implementation. Moreover, firm owners influence the managers’ discretion in strategic change to secure their objectives, e.g., by establishing governance structures. Consequently, it is not surprising that block holder ownership (e.g., Bethel and Liebeskind 1993; Hoskisson et al. 1994), managerial ownership at foundation (Boeker 1989), and changes in ownership after the foundation (Goodstein and Boeker 1991), influence strategic change (Boeker 1989; Goodstein and Boeker 1991). In addition, boards of directors influence strategic change through their own range of experiences as well as by selecting CEOs with certain experience profiles (Westphal and Fredrickson 2001; Zhu et al. 2020). Despite these insights, Müller and Kunisch (2018) call for further research on the influence of owners and ownership characteristics on strategic change.

Second, strategic change is seen as an action to correct a misfit or a shift away from an environmental equilibrium. Thus, the need for strategic change can be triggered by various incidents that cause a shift in equilibrium, for example, increasing volatility (Fombrun and Ginsberg 1990; Gordon et al. 2000), regulations (Smith and Grimm 1987), or new technologies (Yetton et al. 1994). Despite the variety of possible strategic change triggers, researchers agree that, on an abstract level, the world changes in two different modes (Meyer et al. 1990): gradual changes, i.e., first-order or continuous changes, which “… occur within a stable system that itself remains unchanged …” (p. 94), and radical changes, i.e., second-order or discontinuous changes, which “… transform fundamental properties or states of the system …” (p. 94) and lead to the emergence, transformation, or decline of an industry in a short time. In gradual change episodes, firms incrementally and continuously track their environment and adjust their strategy to remain in equilibrium (Meyer et al. 1990). In contrast, radical change episodes require firms to undergo rapid, frame-breaking transformations; in other words, the firm must shift towards a new configuration or archetype to return to equilibrium and correct the misfit with its environment (Amis et al. 2004). Generally, firms undergo relatively long gradual change episodes, punctuated by revolutionary environmental change episodes that require radical strategic change (Tushman and O’Reilly III 1996). In such radical change episodes, “… survival or selection goes to those species with the characteristics needed to exploit the new environment.” (Tushman and O’Reilly III 1996, p. 12).

**Fig. 1:**
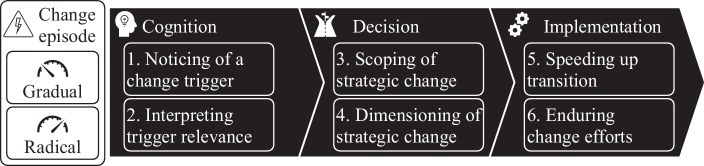
Strategic Change Process

Third, strategic change is a process that proceeds from initiation, after the cognition of a change trigger and a strategic change decision, to implementation (Kunisch et al. 2017) (compare Fig. 1). Strategic change is initiated when actors in a firm become aware of a need or opportunity for change based on their cognition of a change trigger (Helfat and Peteraf 2015; Johnson 1992; Rajagopalan and Spreitzer 1997) and make a strategic change decision that must be appropriate given the environmental predeterminism (Müller and Kunisch 2018). Cognition follows from information seeking to *notice* a change trigger and *interpretation* that creates awareness of a need or opportunity for change (Thomas et al. 1993). According to the attention-based view of the firm (e.g., Ocasio 1997), the cognition of a need for strategic change results from organizational attention (Ocasio et al. 2018). Following cognition, managers need to appropriately *scope* strategic change and decide on the degree to which they delineate the business model and adapt firm boundaries (Teece 2007). Within the decided scope of strategic change, managers need to decide on an appropriate *dimensioning* or magnitude of the strategic change (Rajagopalan and Spreitzer 1997). Following the initiation of strategic change, the changes must be implemented (Kunisch et al. 2017; Stouten et al. 2018) with a certain implementation *speed* (König et al. 2013; Lieberman and Montgomery 1988) and *endurance* (König et al. 2013). Existing research suggests that implementation has a crucial influence on the performance effect of strategic change (e.g., Kunisch et al. 2017).

### Familiness of the Firm

Many researchers have suggested that, globally, the most common type of business firm across size categories is the family firm (e.g., Faccio and Lang 2002; La Porta et al. 1999). The distinctiveness of family firms results from families’ involvement in ownership, management, or control (e.g., Chrisman et al. 2005; Pearson et al. 2008) and the pursuance of unique family objectives (e.g., Chrisman et al. 2012; Gómez-Mejía et al. 2007). According to the resource-based view, the distinctiveness of family firms creates unique, path-dependent resources: the so-called familiness of a firm (e.g., Habbershon et al. 2003; Pearson et al. 2008; Habbershon and Williams 1999; Sirmon and Hitt 2003). According to the definition used by Barney (1991), resources are assets, capabilities, organizational processes, firm attributes, and so on controlled by a firm that enable it to conceive of and implement strategies that improve its efficiency and effectiveness. In the context of family firms, however, familiness may have both positive and negative effects depending on the level and characteristics (Habbershon et al. 2003; Weismeier-Sammer et al. 2013).

According to Habbershon et al. (2003), the formation of familiness is motivated by the systemic vision of the familial coalition. While Habbershon et al. initially focused on transgenerational wealth creation, Chrisman et al. (2003) extended this notion by suggesting that not only wealth creation but also the pursuance of noneconomic objectives incentivizes the formation of familiness. For example, families provide managerial human capital via a family CEO or more subtly by selecting the top management team to pursue family control and influence over the firm and strategic decisions (e.g., Berrone et al. 2012). Moreover, family firms can develop social capital—one of the most important resource bundles in the family firm context, as outlined below—because they care more about their social ties to their stakeholders and can develop patient financial capital due to their dynastic intentions for the firm and their investments (Berrone et al. 2012).

**Fig. 2:**
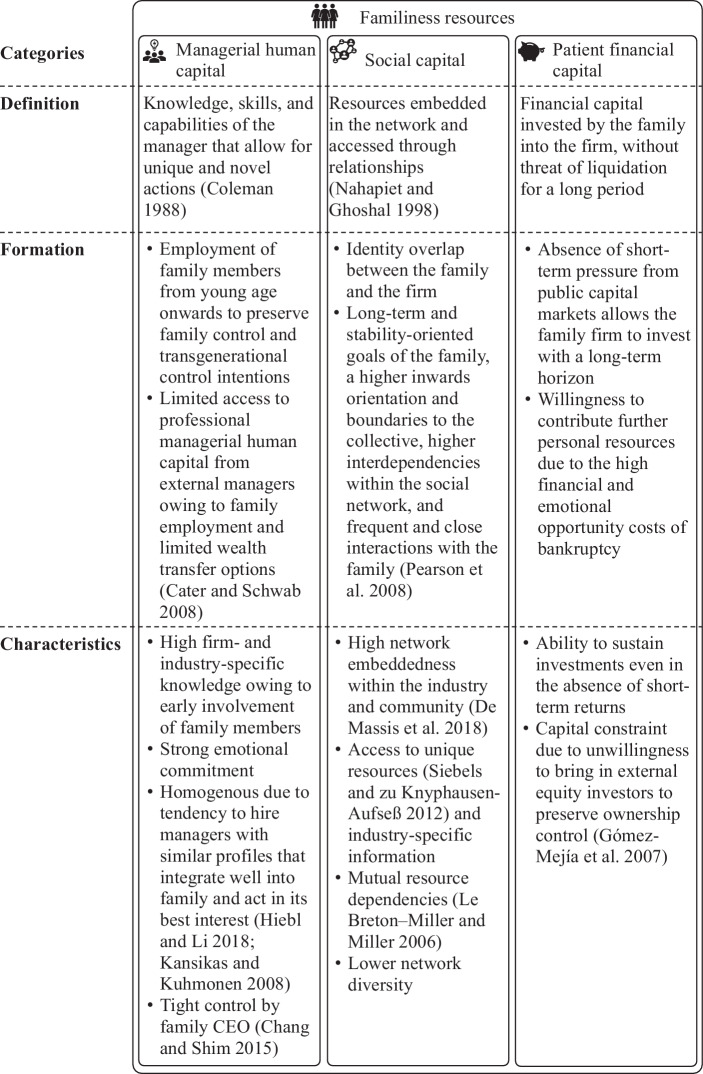
Categories, Definition, Formation, and Characteristics of Familiness Resources

Familiness can be subdivided into resource bundles, including, inter alia, human, social, and patient financial capital (Sirmon and Hitt 2003). For example, reliance on family members in the top management may create deep path-dependent knowledge assets; however, it can also cause a lack of heterogeneity (Sirmon and Hitt 2003). Even if a family hires an external manager, it may have a tendency to hire managers with similar profiles that integrate well into the family and act in its best interest (Hiebl and Li 2018; Kansikas and Kuhmonen 2008), thus increasing the homogeneity of the managerial human capital. Besides managerial human capital, social and patient financial capital are two resource bundles that have gained attention from the scholarly community (e.g., Kano et al. 2020; Pearson et al. 2008; Sirmon and Hitt 2003). The definition of each resource bundle, its formation, as well as its potential characteristics are further described in Fig. 2, primarily based on a synopsis presented by Sirmon and Hitt (2003)—one of the most cited papers in family firm research (Chrisman et al. 2013)—and supplemented by additional scholarly insights.

Even though these resource bundles are commonly used to specify familiness, family firms can differ significantly in terms of characteristics and configurations (Chua et al. 2012; Nordqvist et al. 2014). To navigate this heterogeneity, we focus on a distinct archetype of the family firm that has gained scholarly attention due, for example, to its resource constraints and innovative capacity (Block and Spiegel 2011; De Massis et al. 2018; Soluk and Kammerlander 2021): the German Mittelstand firm. German Mittelstand firms are small- to medium-sized companies that are typically owned and predominantly managed by a family (Decker and Günther 2017; Pahnke and Welter 2019). The resulting family involvement (Chua et al. 1999) has a major influence on such behaviors as decision making or strategy (e.g., Bauweraerts et al. 2019; Casillas et al. 2018; Huybrechts et al. 2013; Miller et al. 2011; Zona 2016). According to De Massis et al. (2018), Mittelstand firms develop industry-specific resources that allow them to focus on a specific niche in which they develop deep expertise and extraordinary efficiencies. Moreover, they have distinct social capital in terms of customer relationships, employee relationships, and community embeddedness. Finally, they possess patient financial capital, resulting in a preference for self-financing and a long-term mindset. Consequently, the Mittelstand firm fulfills many of the distinctiveness criteria suggested by, for example, Sirmon and Hitt (2003). Despite its predominance in Germany, the archetype of the Mittelstand firm can also be found in other national contexts (e.g., Pahnke and Welter 2019; Simon 1996), which extends the relevance of our findings beyond German Mittelstand firms.

Despite the relevance of the RBV-rooted familiness concept for the research field, we want to acknowledge that there are other influential theoretical underpinnings used to explain the distinctiveness of family firms. One important stream of research which has emerged from behavioral agency theory suggests that the pursuance of noneconomic objectives of the family influences the corporate objective function and decision making in family firms. This influence of noneconomic objectives as a determinant in decision making has been conceptualized by family firm researchers under the socioemotional wealth (SEW) umbrella (e.g., Gómez-Mejía et al. 2007), suggesting that “… gains and losses in SEW represent the pivotal frame of reference that family-controlled firms use to make major strategic choices and policy decisions.” (Berrone et al. 2012, p. 259). Generally, family firms balance economic objectives with noneconomic objectives in decision making (Alessandri et al. 2018; Gomez-Mejia et al. 2018), including family control and influence, identification with the firm, binding social ties, emotional attachment, or the renewal of family bonds (Berrone et al. 2012). While family firms usually prefer noneconomic objectives under normal operating conditions, their willingness to compromise increases with perceived hazard to the firm (Gomez-Mejia et al. 2011; Gómez-Mejía et al. 2007; Zellweger and Astrachan 2008). Even though we use familiness and its underlying resource bundles as the guiding framework for this article, we refer to SEW especially throughout the formulation of the propositions on decision making to acknowledge the concept’s importance for family firm research.

### Familiness and Strategic Change

While evidence from both family firm (e.g., Brunninge et al. 2007; Chirico and Salvato 2008; Drozdow and Carroll 1997; Ogbonna and Harris 2001; Ward 1997) and strategic change research (e.g., Bethel and Liebeskind 1993; Boeker 1989; Bohman and Lindfors 1998; Bourgeois III 1984; Goodstein and Boeker 1991) is conclusive on the influence of family involvement in ownership and management on strategic change, there is ambiguity about whether familiness facilitates or impedes strategic change.

Family firms are not only the most common form of business ownership, but also reach remarkable ages. Thus, family firms appear to be successful in achieving their key objective: sustaining the firm and legacy for future generations. Moreover, many scholars have suggested that family firms outperform nonfamily firms (e.g., Anderson and Reeb 2003a; Lee 2006; Martínez et al. 2007; Maury 2006). These observations imply that family firms are an especially viable and adaptable type of business ownership. For example, scholars have suggested that family firms are good at continuous, less disruptive innovation in their current niches (Zellweger et al. 2010). When family firms engage in strategic change, such as divestitures (Feldman et al. 2016), diversification (Adhikari and Sutton 2016) or acquisitions (André et al. 2014; Caprio et al. 2011), they are often more successful than their nonfamily firm counterparts.

However, many scholars draw a contradicting picture of family firm adaptability. They suggest that loyalty to family traditions and employees often causes resistance to strategic change (Chirico and Salvato 2008; Drozdow and Carroll 1997; Ogbonna and Harris 2001; Ward 1997). Families may perceive a high “… affinity to the business grandad built” (Harris et al. 1994, p. 162), potentially isolating them from their external environment (Brunninge et al. 2007), causing a negligence of changes away from the status quo (Eddleston et al. 2012) and encouraging successors to stay in the current strategy (Mitchell et al. 2009). Family firms often describe themselves as less aggressive to protect core family values and their niches (Zellweger et al. 2010); they tend to avoid drastic changes to preserve family harmony (Salvato et al. 2010). This preference for noneconomic objectives may thus inhibit strategic change (Williams Jr et al. 2018). Instead, Mittelstand firms tend to prefer organic growth strategies with limited need to dilute familiness resources. In internationalization, for example, they can leverage existing resources in new markets (De Massis et al. 2018). Moreover, family firms that are acquired, for example, by private equity funds (Scholes et al. 2010), are likely to change their strategy following the acquisition, indicating a latent need for strategic change that is impeded by family ownership (Scholes et al. 2009).

Research has shown the distractive effects of familiness on strategic change by studying different types of strategic change. For example, family ownership and family involvement in management decrease the likelihood of divesting assets (Caprio et al. 2011; Chi-Nien and Xiaowei 2008; DeTienne and Chirico 2013; Feldman et al. 2016) or diversifying (Aktas et al. 2016; Anderson and Reeb 2003b; Gomez-Mejia et al. 2010; Schmid et al. 2015). One of the most prominent types of strategic change that has received widespread attention from family firm researchers is technology adoption and innovation. For example, König et al. (2013) proposed that, due to emotional ties to existing assets and rigid mental models, family firms are slower in recognizing disruptive technological changes, less aggressive in their response thereto and, ultimately, less flexible in implementing such changes to protect familiness resources and their noneconomic benefits (Chrisman et al. 2015; De Massis et al. 2015; Kammerlander and Ganter 2015; Schäfer et al. 2017). The firms’ management seldom question existing technologies, are unwilling to engage in external partnerships, and are predominantly inward-looking, causing them to focus on product innovation (Alberti and Pizzurno 2013).

Furthermore, existing research suggests that familiness influences all the steps in the strategic change process. Family firms often have a performance bias in that they interpret their performance as too positive, which leads to a rigid commitment to old strategies (Ogbonna and Harris 2001). They often revert to family narratives to justify a decline as only temporary (Salvato et al. 2010). Additionally, family conflict can divert attention from screening for a need for change (Ward 1997). For example, family firms are less likely to acquire new technologies in response to underperformance (Kotlar et al. 2013). Thus, familiness influences the cognition of a need or opportunity for change.

Furthermore, familiness may inhibit the strategic change decision. For example, patient financial capital and the resulting avoidance of external financing sources constrains strategic decision making (Harris et al. 1994), innovation (Pukall and Calabrò 2014; Schäfer et al. 2017), or internationalization (Yenn-Ru et al. 2009). In the Mittelstand firm, in particular, the financing constraints may lead the firm to sacrifice opportunities during economic booms but increase resilience during economic crises (De Massis et al. 2018). In the context of technology, a family’s goal preferences strongly influence whether a new technology is adopted (Kammerlander and Ganter 2015). In a crisis, however, families tend to overcome the differences in members’ objectives and invest everything to protect the traditional business (Cater and Schwab 2008), for example, by engaging in diversification decisions (Aktas et al. 2016), increasing investments in new technologies (Souder et al. 2017), or becoming more willing to take risks (Chrisman et al. 2015; Gomez-Mejia et al. 2010).

Ultimately, the characteristics of family firms have been found to influence the implementation of innovation (Gudmundson et al. 2003). For example, family firms can benefit from the lower formalization and lower political resistance that result from the families’ authority in the firms in the implementation of new technologies (König et al. 2013). Moreover, the exceptional employee relations in family firms enhance employee commitment, willingness, and motivation to implement innovation (Bennedsen et al. 2007; Cassia et al. 2012; De Massis et al. 2018). In addition, family firms can activate their social network to enhance the implementation of innovation projects by leveraging, for example, the strong relationships with existing suppliers or universities (Grundström et al. 2011).

Summarizing the above, we conclude that, from both family firm and strategic change research perspectives, a more detailed concept of how familiness, as an ownership characteristic, influences strategic change is required to explicate the ambiguity outlined in this section. In addition, we have pointed out how the influence of familiness can vary with change episodes, e.g., regarding the adoption of new technologies (König et al. 2013). Thus, a conceptualization of the influence of familiness on strategic change should account for the different strategic change requirements in gradual and radical change episodes (e.g., Meyer et al. 1990). Finally, family firm literature suggests that, to truly conceptualize the effect of familiness on strategic change, a differentiation of its influence on the different stages of the strategic change process is required.

## Toward a Model of Strategic Change in the Family Firm

As suggested by Barney (1991), in his definition, resources have to be appropriated in a strategic context to be valuable. Resources are thus closely linked to strategy, and their effects must be considered within a firm’s broader strategic context. Furthermore, reconfiguring resources as part of strategic change is especially relevant in fast-moving business environments (Nason and Wiklund 2018), in other words, in radical change episodes. However, firm resources such as managerial human capital (Helfat and Martin 2015) are intertwined with the capability to adapt them to changing environments (Teece and Pisano 2003; Teece 2014). This means that resources not only are affected by strategy, as suggested by Barney, but also affect strategizing itself.

**Fig. 3:**
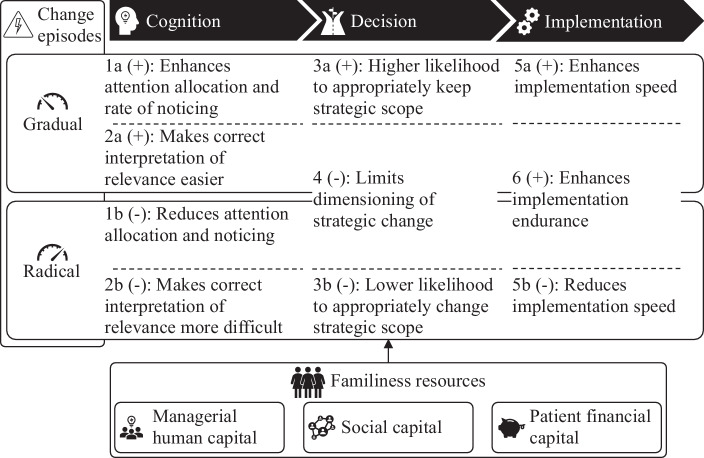
Overview of Propositions

Accordingly, we develop propositions on how familiness influences the presented steps of the strategic change process. For each of the six mechanisms of the strategic change process defined in the previous section (i.e., noticing of a change trigger, interpretation of its relevance, scoping of strategic change, dimensioning of strategic change, speeding-up transition, and enduring change efforts, compare Fig. 1), we discuss the influence of familiness by looking at its most influential features. The propositions show that familiness has both supporting and limiting effects on the strategic change process. Whenever necessary, we differentiate between gradual and radical change episodes in our propositions. A summary of the propositions is provided in Fig. 3.

### Familiness and the Cognition of Gradual and Radical Change Triggers

Following the framework of Thomas et al. (1993), we define cognition as information seeking and interpretation that creates an awareness of a need or opportunity for change. Successful cognition requires *attention allocation* to notice a change trigger as a precursor of a change episode (Ocasio 1997). Due to their bounded rationality, managers can only allocate attention to a limited number of change triggers based on 1) the individual focus of attention, 2) the situational context, and 3) the organizational context.

First, Kiesler and Sproull (1982) outline research on social cognition and suggest that managers differ in terms of how they focus their attention. They argue that managers only attend to information that they can directly link to their individual aspirations. Moreover, information with strong subjective signaling power to the manager can divert attention from relevant change triggers. Consequently, managers tend to focus on information that reinforces their worldviews and self-understanding, especially when they feel personally invested in a situation (Kiesler and Sproull 1982). These individual differences in focus can partially be explained by a manager’s experience and knowledge (Cornelissen and Werner 2014; Helfat and Peteraf 2015). We mentioned previously that managers in family firms tend to obtain higher firm-specific experience and knowledge and to have distinct aspirations that resulted from their noneconomic objectives for the firm. For example, familiness causes strong emotional commitment, culminating in a family identity that can be strongly intertwined with a firm’s business activities, such as being a “brewing family” (Habbershon and Pistrui 2002). Consequently, family managers will be more receptive to gradual change triggers with an unambiguous effect on the business activities and, thus, on the family identity. In contrast, radical change triggers with an ambiguous influence on these factors are more likely to be drowned by the signaling power of gradual change triggers or to be ignored by family managers who wish to avoid the realization that the relevance of the legacy business activity as an essential element of family identity diminishes. For example, managers in family firms may ignore financial performance deviations, such as below-target (DeTienne and Chirico 2013) or decreasing performance (Salvato et al. 2010; Sharma and Manikutty 2005), as long as noneconomic aspirations can be sustained.

Second, the situational context shapes attention allocation (Ocasio 1997), for example, via information provided from a social network (Adner and Helfat 2003; Helfat and Martin 2015). Network theory argues that the value of this information depends on the strength of social network ties (Granovetter 1983). Strong social ties within a network improve the availability of information and increase the stakeholders’ motivation to collaborate. Weak social ties provide access to information that is more distant to common knowledge, making them a valuable source of information on radical change triggers that start to evolve outside of established industries. Due to their tendency to avoid external dependencies (e.g., Gómez-Mejía et al. 2007), family firms are more likely to develop social networks with similar types of firms (Basly 2007; Pukall and Calabrò 2014). Moreover, family firms tend to develop strong social ties to their stakeholders as part of their social capital (Pearson et al. 2008). This deep embeddedness in a social network, which is characterized by strong social ties, creates a situational context in which attention is predominantly focused on gradual developments within an industry. However, the relative scarcity of weak ties in the network reduces access to more distant information on radical change triggers.

Third, the organizational context influences the breadth of attention allocation, for example, via the distribution of decision-making authority within a firm (Ocasio 1997). In family firms, decision power is typically kept within the family because of the desire to protect the family’s control (Berrone et al. 2012; Gómez-Mejía et al. 2007). External managers are often selected with a bias toward reinforcing traditions (Kansikas and Kuhmonen 2008) and act in the interest of the family (Hiebl and Li 2018), creating a homogeneous group of managers with often lower information diversity (Xiaowei and Chi-Nien 2005). Indeed, research has shown that certain management team characteristics, e.g., shorter organizational tenure or higher specialization diversity, increase the propensity to change a firm’s corporate strategy (e.g., Boeker 1997; Wally and Becerra 2001). The smaller and more cohesive group of managers in the family firm, in contrast, will likely focus on gradual change triggers that are omnipresent in their daily work instead of embracing different, more diverse foci of attention. Indeed, research has shown that family managers are often preoccupied with day-to-day operations, failing to focus on long-term change initiatives (Pal et al. 2014). Additionally, family shareholders have been found to be more patient and to exert weaker governance mechanisms, such as performance evaluations (Gersick 1994), thus reducing the pressure on managers to broaden their attention allocation.

In summary, the specificity of managerial human and social capital creates a strong focus of attention on gradual change triggers that are perceived to be most salient. An absence of weak social ties with access to more distant information reduces the attention allocated to radical change triggers. A smaller circle of decision-makers combined with weaker shareholder pressure and governance further reduces the breadth of attention allocation. We thus propose the following:

*Proposition 1a: Familiness enhances the attention allocation to gradual change triggers and, consequently, increases the corresponding rate of noticing.*

*Proposition 1b: Familiness reduces the attention allocation to radical change triggers and, consequently, decreases the corresponding rate of noticing*.

Once a change trigger has caught decision-makers’ attention, they interpret its relevance before initiating strategic change. However, “… individuals may over-, under-, or mis-estimate the importance of particular environmental features as causal agents …” and falsely interpret especially more discrepant change triggers (Kiesler and Sproull 1982, p. 552). We argue that familiness influences *interpretation* through 1) cognitive frames applied by the decision-makers, 2) group dynamics, and 3) performance thresholds.

First, interpretation is guided by the cognitive frames that are applied by managers. Cognitive frames or knowledge structures help managers to evaluate information, interpret its relevance (Kiesler and Sproull 1982), and evaluate the consequences for the firm. If managers use inappropriate cognitive frames, they may underestimate the relevance of change triggers. Generally, cognitive frames result from a manager’s knowledge and experience (Cornelissen and Werner 2014). In the context of radical change triggers, correct interpretation requires distinct knowledge structures (Kiesler and Sproull 1982) that managers can derive from previous experience in other changing organizations (Helfat and Martin 2015). Owing to the cohesion of managerial human capital and the strong exposure of family managers to the firm, family firms tend to possess industry- and company-specific instead of universal knowledge structures (Sieger et al. 2011); when evaluating especially more ambiguous radical change triggers against these established knowledge structures, their relevance may be underestimated. Moreover, instead of changing the knowledge structures to correct this misinterpretation (Rajagopalan and Spreitzer 1997), family managers revert to the cognitive frames that helped them overcome past crises, which often leads to a misinterpretation of, for example, declining performance (Salvato et al. 2010). In other words, the composition of managerial human capital in family firms creates a reliance on firm-specific knowledge structures, potentially obscuring the interpretation of radical change triggers.

Second, restricting interpretation to a small and cohesive group risks defective judgment because individuals seek concurrence while personal attitudes tend to converge (Janis 1972). Managers may, therefore, risk adopting the interpretation of, for example, a strong family manager instead of critically questioning it. A key objective of family managers is to avoid conflict (Sharma and Manikutty 2005), which increases their consensus orientation (Cater and Schwab 2008; LaRocca and De Feis 2015). Consequently, they will be less likely to challenge each other’s interpretations if such action threatens the harmony within the management team or with the shareholders. As we previously outlined, the interpretation of a radical change trigger often conflicts with the resulting self-understanding of the family. To avoid the resulting controversy and potential conflict in the group of managers and shareholders, we expect familiness to increase the convergence of interpretations.

Third, managers may fail to correctly interpret environmental change if they perceive it as irrelevant compared to their own point of reference or threshold (DeTienne and Chirico 2013; Kiesler and Sproull 1982). A key factor that drives a manager’s performance threshold is the perceived threat of employment loss with the resulting damage to the manager’s reputation in the employment market. To avoid this threat, managers often initiate hasty responses, for example, to declining performance (Morrow Jr et al. 2007). For family managers, their perceived employment guarantee in the firm reduces the significance of this threat, thus reducing the pressure to immediately address environmental changes to satisfy investors. However, the strong association with the firm elevates the emotional commitment while the transgenerational vision increases the motivation to overcome roadblocks (Bennedsen and Foss 2015) and remain competitive (Chirico and Salvato 2008) to preserve the family legacy. Therefore, causing the firm to fail would lead to more significant emotional stress for family managers. Furthermore, the family would lose the patient financial capital and time invested in the firm (Sirmon and Hitt 2003). Overall, these considerations are likely to outweigh the lower threat of reputational damage in the employment market; however, their full effect only manifests when firm survival is threatened, potentially causing an interpretation delay.

Based on the above, we conclude that familiness influences the interpretation of change triggers. Cohesive managerial human capital creates industry- and firm-specific cognitive frames that enhance the interpretation of gradual change triggers. However, the absence of universal knowledge structures constrains the interpretation of radical change triggers. The group dynamics of a smaller circle of decision-makers will lead to cohesion of interpretations. If, however, the cognitive frames allowed the decision-makers to interpret a change trigger, the willingness to protect the family legacy would raise the perceived relevance. We thus propose the following:

*Proposition 2a: Familiness makes the correct interpretation of the relevance of gradual change triggers easier and, consequently, increases the response rate.*

*Proposition 2b: Familiness makes the correct interpretation of the relevance of radical change triggers more difficult and, consequently, decreases the response rate.*

### Familiness and the Strategic Change Decision

After cognition has created the awareness of a need or opportunity for strategic change, it must be decided how to respond. In radical change episodes, firms are often required to scrutinize both business model and enterprise boundaries and, thus, challenge their strategic scope. Gradual change episodes, in contrast, require a focus on improving the competitiveness of the current business model within the existing industry. When deciding on the *scope* of strategic change, decision-makers balance 1) the perceived economic and 2) noneconomic value of existing resources, often referred to as SEW in the family firm context (e.g., Gómez-Mejía et al. 2007).

First, familiness affects the economic value of existing resources. Generally, firms focus on improving the use of existing resources rather than exploring new ones (Kraatz and Zajac 2001). Familiness resources are often developed over generations with the objective of passing them on and enhancing them from generation to generation. Consequently, they are highly path dependent and very specific to the firm scope and context. For example, managerial human capital in a family firm is often specifically tailored to the current business context, especially if the managers spend most of their career in the firm. This deep tacit knowledge is valuable for the firm and—due to its path dependency—difficult for competitors to copy, making it a source of competitive advantage. This effect is similar for social capital, which is characterized by strong social ties, high proximity, and trust, making it again a valuable source of competitive advantage for the family firm. Changing the strategic scope of the firm would mean that the family relinquishes the scope-specific competitive advantage of familiness, thus increasing the economic opportunity costs of such a change and the economic incentive to remain in the current strategy.

Second, familiness also affects the noneconomic value of existing resources. Families value the self-conception that results from loyalty to legacy and traditions (Ogbonna and Harris 2001; Ward 1997). A reinvention of the business model by changing the strategic scope of a firm would conflict with the family’s self-conception. For example, a divestment will lead to a break in the family identity (Livengood and Reger 2010) and may be perceived as a personal failure (Salvato et al. 2010). In addition, a change in strategic scope would require new managerial resources with expertise and familiarity with the new business context. To fill this expertise gap, the family would have to inject external expertise and knowledge, thus diluting the family managers’ control (Gomez-Mejia et al. 2010) and harming the SEW of the family. Prior research has found that family firms tend to avoid diversification decisions due to a lack of knowledge and expertise (Aktas et al. 2016). Furthermore, a shift in a business model might require a shift in general human resources, necessitating replacement or transformation of the current workforce. Such a break would weaken the social ties to the current employees, suppliers, and even customers, potentially, and could harm the family’s social status within its network.

Overall, we argue that, when deciding on the type of strategic change, family firms decide within different parameters. The higher perceived emotional and economic value of existing resources encourages a limited scope for strategic change, which we deem to be particularly appropriate in the context of gradual change episodes. However, in a radical change episode, these parameters hamper the process of changing the strategic scope, consistent with the research on the role of SEW as a key noneconomic family objective in decision making (e.g., Berrone et al. 2012; Gómez-Mejía et al. 2007; Gomez-Mejia et al. 2018). We thus propose the following:

*Proposition 3a: Once a gradual change episode is recognized, familiness enhances the likelihood of appropriately keeping the strategic scope.*

*Proposition 3b: Once a radical change episode is recognized, familiness reduces the likelihood of appropriately changing the strategic* scope.

Next, a firm needs to allocate resources to the selected strategy and define the *magnitude* of the strategic change. In the following paragraphs, we connect familiness to the dimensioning of strategic change based on the insights presented in the previous section and focus on 1) the influence of the perceived value and complement this view by investigating 2) access to resources.

First, high-magnitude strategic change can conflict with family harmony and social ties to stakeholders. The greater the magnitude of the change, the greater the potential conflict with the family legacy. Consequently, high-magnitude strategic change is a potentially controversial decision and may be avoided to preserve family harmony (Salvato et al. 2010). The perceived commitment toward stakeholders as part of social capital creates an additional burden on high-magnitude strategic change as it requires greater adaptation by the stakeholders. For example, a shift in regional focus may require a relocation of capacities and employees. In such cases, a strong loyalty to employees, social responsibility, and the protection of family reputation (Block 2010) can limit what family firms expect from stakeholders, especially their employees.

In addition, patient financial capital influences access to resources. On one hand, it increases the willingness to accept short-term losses to pursue substantial investments that create long-term benefits. On the other hand, familiness limits the amount of resources that can be committed to strategic change. We have described how patient financial capital limits access to external capital markets (Sirmon and Hitt 2003) and creates additional burdens on debt utilization (Pukall and Calabrò 2014; Williams Jr et al. 2018) due to the corporate objective of sustaining ownership control. The longevity of current investments may further constrain capital access by making it more difficult for a firm to shift resources. The resulting lack of capital can become a constraint on strategic decision making (Harris et al. 1994) by limiting the firm’s ability to commit sufficient resources to high-magnitude strategic change, for example, in the context of disruptive technologies (König et al. 2013). We thus propose the following:

*Proposition 4: Familiness negatively influences the magnitude, that is, the dimensioning, of strategic change during both radical and gradual change episodes*.

### Familiness and Strategic Change Implementation

After the decision, a firm needs to implement the strategic change. We first analyze the influence of familiness on implementation *speed*, which, we believe, is mainly driven by 1) formalization, 2) stakeholder commitment, and 3) the need to develop resources. We subsequently analyze the influence of familiness on implementation endurance.

First, family managerial human capital can lead to the centralization of decision-making authority, which is generally considered harmful to implementation speed due to the isolation of the management team (Teece 2007). In contrast, family firms are also often characterized by lower formalization that can, for example, increase the adoption speed of discontinuous technologies (De Massis et al. 2015; König et al. 2013). In other words, the lower formalization reduces bureaucracy and increases overall implementation speed. However, the lower formalization may be accompanied by weaker external governance that results from patient financial capital. This can lead to less formal implementation progress monitoring and increase the tolerance for a lower implementation speed.

Second, familiness affects commitment to strategic change. Social capital in the family firm helps to foster employee commitment, which is a key driver of implementation success (Stouten et al. 2018) and increases employee willingness to contribute to the implementation. Lionzo and Rossignoli (2013) found that the strong informal networks among a firm’s internal stakeholders increased social interaction within the firm, again increasing implementation speed. However, social capital also constrains implementation speed owing to a firm’s commitment to its stakeholders. This commitment may induce the family to reduce speed to allow its stakeholders a smooth adaptation. For example, family firms are slower in replacing leading employees owing to their strong loyalty (Lynn and Rao 1995).

Third, strategic change that involves a shift in the strategic scope of a firm can create a need to build new resources. Family managerial human capital, with its objective of preserving family control, may create a barrier to acquiring external resources to avoid new dependencies and loss of family control as part of SEW. For example, the desire to preserve family control may negatively influence the decision to acquire new technologies (Kotlar et al. 2013; Souder et al. 2017) or to internationalize (Pukall and Calabrò 2014) because of the resulting need for external expertise and partners. Given this reluctance to incorporate external expertise, the firm must develop the required resources internally, significantly reducing implementation speed.

Synthesizing the arguments presented above, we conclude that familiness has a mixed impact on implementation speed. In a gradual change episode, a firm will benefit from lower formalization and a higher commitment of human and social capital; meanwhile, there is a lower need to acquire external resources. In contrast, in a radical change episode, the desire to protect family control hinders the acquisition of external resources, necessitating lengthy internal development. Moreover, the higher centralization of decision making and the lack of short-term pressures increase the tolerance for slow implementation. The protection of stakeholder interests can further slow implementation. We thus propose the following:

*Proposition 5a: Familiness enhances the implementation speed of strategic change in a gradual change episode.*

*Proposition 5b: Familiness reduces the implementation speed of strategic change in a radical change episode.*

We next investigate how familiness influences implementation *endurance*. For this purpose, we analyze the influence of familiness on a firm’s 1) commitment of managers and stakeholders and 2) financial resources required for implementation.

First, family managers’ commitment positively influences employee behavior (Zahra et al. 2008). Consequently, when a firm faces drawbacks, the commitment of family managers will enhance collective commitment and therefore implementation endurance. For example, research has shown that, among other factors, decision-makers’ stability helps sustain motivation and unity during crises (Wilson et al. 2013). In addition, family social capital enhances the resilience of the resource commitment from stakeholders by, for example, increasing the longevity of business deals (Harris et al. 1994) as well as the general willingness of employees to contribute, as outlined previously. This means that stakeholders are more likely to sustain their commitment despite setbacks. For example, family firm stakeholders are likely to be more supportive and altruistic in times of crisis to ensure firm recovery (Cater and Schwab 2008).

Furthermore, implementation endurance is influenced by patient financial capital. Particularly in the case of high-magnitude strategic change, the financial results may not be immediately visible whereas the implementation costs will remain high. Patient financial capital with weaker short-term financial pressures allows a firm to invest its funds freely and flexibly (Dailey et al. 1977) and, thus, sustain the investment even in the absence of visible financial returns (De Massis et al. 2018). As mentioned, however, family patient financial capital can also create a financing constraint: the firm can run out of the funds required to sustain implementation, especially if unplanned additional investments are required. However, we argue that the willingness of the family to provide additional financial resources as part of patient financial capital—also referred to as survivability capital (Sirmon and Hitt 2003)—allows a firm to be more resilient when setbacks arise during implementation.

In conclusion, patient financial capital enables a firm’s persistence during implementation despite the absence of short-term returns or in times of crisis. In addition, the strong commitment of human and social capital enhances the firm’s resilience during implementation even when setbacks arise. We thus propose the following:

*Proposition 6: Familiness positively influences endurance during the implementation of strategic change.*

## Discussion

To summarize the previous section, we conclude that, while familiness affords family firms an edge in gradual change episodes, it can become a liability in radical change episodes, thus providing a differentiated picture of the positive and negative effects of familiness (Weismeier-Sammer et al. 2013). More precisely, based on the propositions put forward above, three tendencies of family firms in strategic change are proposed: 1) while familiness enhances the cognition of gradual change triggers, it also increases the likelihood of either missing or falsely interpreting radical change triggers; 2) while familiness creates a decision tendency that is beneficial in gradual change episodes, it negatively affects the likelihood of changing strategic scope and engaging in high-magnitude strategic change that may be required in radical change episodes; and 3) while familiness supports implementation endurance and can be beneficial in implementing gradual change, it becomes constrictive in the implementation of radical change since it reduces the implementation speed and increases the risk of stubbornly implementing insufficient changes.

The proposed tendencies facilitate several presumptions on the performance effect of strategic change on family firms. Understanding performance differences between family and nonfamily firms has drawn widespread attention from the scholarly community (e.g., Anderson and Reeb 2003a; Lee 2006; Mazzi 2011; Rutherford et al. 2008; Ward 1997). However, the empirical findings remain contradictory. Moreover, there is no consensus in the strategic change literature on the moderators of the performance effects of strategic change (e.g., Müller and Kunisch 2018), including on the potential effect of family ownership (e.g., Zahra et al. 2008).

We suggest that the three tendencies due to familiness enhance the cognition, decision, and implementation of strategic change in gradual change episodes. Here, a family firm incrementally adapts to environmental changes. Therefore, we expect familiness to positively influence the firm’s performance during, for example, continuous, nondisruptive innovations in the current niche (Zellweger et al. 2010). This is consistent with the findings by Anderson and Reeb (2003a), who—based on a study of S&P 500 firms—found that family firms tended to outperform their peers in the nonfamily sector. By contrast, in a radical change episode, the equilibrium within an established industry changes significantly within a short time (Hildebrandt et al. 2018). In this case, we put forward that familiness tends to delay cognition, hinder the decision to engage in the strategic change of the required scope and magnitude, and reduce the implementation speed. Consequently, family firms would be at a disadvantage compared to nonfamily firms. Even if an industry recovered to stability (Meyer et al. 1990), familiness might no longer be a source of competitive advantage because its resources would have become less valuable in the new equilibrium. Consequently, we would expect to find a more negative performance by family firms compared to nonfamily firms in and after radical change episodes.

Other studies suggest that family firms are generally less likely to fail (Wilson et al. 2013). A possible explanation for this observation is that, even if a family firm responds late or insufficiently in a radical change episode, it is sustained by its survivability capital, albeit at the cost of competitive advantage and economic rent (DeTienne and Chirico 2013). This is consistent with the finding that two-thirds of family firms that survive over a 60-year period do not grow over that time (Ward 1997).

In the worst case, insufficient strategic change creates a risk of business failure (Haveman 1992; Meyer et al. 1990). A prominent line of thought in family firm research suggests that family firms face a significant risk of business failure following a generational transition (e.g., Aronoff 2004; Vallejo 2008; Ward 1997). However, the argument presented here offers an alternative explanation for such failures. While the founding generation is likely to have seized an opportunity in a new or growing industry, typical industry lifecycles suggest that subsequent generations may face a need for radical change. As we have proposed, the occurrence of such a radical change episode increases the likelihood of business failure, owing to the three tendencies of familiness. In this case, failure may thus be caused by inadequate strategic change, not by the succession event as such. Moreover, the occurrence of radical change episodes may be one factor that contributes to the lifespan variance between family firms outlined by Ciravegna et al. (2020).

## Limitations and Areas for Future Research

In this article, we have proposed that familiness affects strategic change. We have further suggested a link between the propositions and firm performance and presented how they can create a threat to firm survival in radical change episodes. However, there may also be a reverse effect of strategic change on familiness formation or depletion. Family firms’ engagement in strategic change may impact not only firm performance but also familiness. For example, a high-magnitude change in strategic scope reduces the value of many elements of familiness, such as social or human capital. However, some elements of familiness, such as patient financial capital or survivability capital, can potentially be sustained, resulting in a very different familiness configuration. In a gradual change episode, in contrast, strategic change reinforces familiness by strengthening the relationship with employees as part of social capital or by further enhancing the firm-specific value of managerial human capital. In other words, our propositions raise the question of whether familiness is also an outcome of strategic change. If this were true, not only family episodes but also strategic change episodes would influence familiness configurations. This implies that not only family variables but also the environmental context may influence the heterogeneity among family firms.

Nevertheless, we are aware that our work does not fully accommodate heterogeneity among family firms, a common limitation in management research (e.g., Narula et al. 2019). We have not accounted for the influence of different institutional or cultural contexts. For example, large family-owned conglomerates in India tend to have wider networks across geographies and industries than their nonfamily peers owing to their ability to compensate for institutional weaknesses (Khanna and Palepu 2000). Other enterprising families may have specialized in the ability to shift resources into the most promising new industries and business activities and may see themselves more as investors (Habbershon and Pistrui 2002). Thus, researchers and practitioners should interpret our propositions in the specific context of the firm being studied.

However, this limitation opens a key area for future research, namely, how family firms can proactively adapt their familiness to overcome the three tendencies proposed here. Family firms that want to ease the constrictive effects of familiness can, for example, adapt their governance, open the firm to external managerial human capital (Brunninge et al. 2007), or redefine the family identity from a family business to a business family (Habbershon and Pistrui 2002; Salvato et al. 2010). In summary, investigating a broad set of firms with different degrees of familiness may contribute to our understanding of how family firms can overcome the tendencies described here to become more effective in strategic change processes.

Another common field in family firm research is the role of family-related events, such as family conflict or succession. In the context of this article, the resulting question is how change triggers and family-related events interact. Specifically, how do distinct family events along the lifecycle of a family firm moderate the influence of familiness on the strategic change process? Existing research has pointed out that family firms tend to be most innovative following a succession (Zellweger et al. 2010). In contrast, other scholars have found that the trend in business direction following a succession is continuity (Grundström et al. 2011). If family conflict occurs, family members working in the firm may not be able to create a viable business plan to change the firm’s strategy (LaRocca and De Feis 2015). In summary, our propositions provide a framework for researchers to investigate the impact of family-related events on strategic change.

The propositions that we put forward in this study require empirical validation, which we suggest should be done either based on longitudinal or decision-related data. Longitudinal studies could investigate the historical evolution of family firms, spanning multiple strategic change decisions and variations in familiness. This could enhance our understanding of how changes in familiness and family-level events affected strategic change, and how family firms sustained their familiness, even following a high-magnitude strategic change. One example of this research setup is the case study of Falck, an Italian family firm that evolved from a steel company into a business group (Salvato et al. 2010). In contrast, decision-related research could investigate the decision-making process for strategic change in family firms in more detail.

Empirical research could focus on a firm or industry. At the firm level, such research could focus on strategic change decisions that were unrelated to each other. In contrast, industry-level research could compare strategic change across multiple firms that faced similar change episodes. This would allow a comparison of the strategic change process between family and nonfamily firms as well as between different forms of familiness. Existing examples of industry-level studies are an analysis of a hospital industry’s reaction to a disruptive regulatory change (Meyer et al. 1990) and the response of Spanish olive mills to market consolidation (Gómez-Mejía et al. 2007). Ultimately, combining data from different industry-level studies allows scholars to consider whether a generic concept of strategic change in family firms based on the differentiation between gradual and radical change episodes (as proposed in this article) is valuable or whether a more granular consideration of industry-level factors is required.

Furthermore, we encourage research to translate the presented propositions into general strategic change research. Although we explicitly link familiness to family firms based on the underlying assumption of the identity overlap between family and firm, several dimensions of familiness can also be found in nonfamily firms. For example, a firm with a strong regional focus may face similar limitations in terms of human and social capital. Firms located in weak institutional environments with weak capital markets may face similar capital constraints. Future research should untangle the propositions presented here to determine how firm and managerial resources affect strategic change, thus advancing, for example, the literature on dynamic managerial capabilities (e.g., Adner and Helfat 2003; Helfat and Martin 2015).

Another pivotal aspect in the translation of our work into general research is a critical examination of the corporate objective function. We concluded that family firms decide within different parameters as opposed to nonfamily firms (Gómez-Mejía et al. 2007) and that those preferences influence, for example, the strategic change decision. Moreover, we discussed how the tendencies of family firms influence firm performance during strategic change. However, we adopted a normative definition of performance that was solely focused on financial implications; indeed, we believe that this financial perspective becomes the more relevant the more the survivability of a company is at risk. If, however, the risk is less severe, the SEW perspective outlined above suggests the possibility of considering other success criteria for change episodes, such as maintaining the workforce or following specific sustainability standards. Moreover, Schwarz et al. (2021) argue that it is always a matter of sensemaking and interpretation whether a change episode has succeeded or failed and that it may well be that failure at one point leads to a successful outcome at another time. We believe that both of these considerations—considering other success criteria that, given assured survival, go beyond a purely financial approach and incorporating a dynamic interpretive perspective on success and failure—can also open up promising avenues for further research.

A final remark on the family itself as an underlying construct of familiness is necessary: In recent years, family structures have experienced changes involving increasingly atypical structures, such as same-sex couples, multigenerational and extended families, and virtual families (Beauregard et al. 2009). These developments may influence family-level events such as family conflicts; however, they may also shape the elements of familiness, for example, by challenging the definition of who is considered a family member or by reshaping family identities. It would be interesting to see how such developments influenced the definition of a family firm, familiness, and, potentially, even the propositions presented in this article.

## Footnotes

1 Based on the publication, “Die ältesten Familienunternehmen Deutschlands” [Germany’s oldest family firms], published by Stiftung Familienunternehmen [Foundation for family firms] in 2019, which can be accessed under the following link: https://www.familienunternehmen.de/media/public/pdf/pressebereich/meldungen/2019/2019-06-06/die-aeltesten-familienunternehmen-deutschlands_2019-06-06_liste.pdf.
